# Growth and development of the placenta in the capybara (Hydrochaeris hydrochaeris)

**DOI:** 10.1186/1477-7827-7-57

**Published:** 2009-06-03

**Authors:** Claudia Kanashiro, Tatiana C Santos, Maria Angelica Miglino, Andrea M Mess, Anthony M Carter

**Affiliations:** 1 Department of Surgery, School of Veterinary Medicine, University of São Paulo, São Paulo, Brazil; 2 Department of Animal Science, State University of Maringá, Paraná, Brazil; 3 Department of Research, Museum of Natural History, Leibniz-Community, Berlin, Germany; 4 Department of Physiology and Pharmacology, University of Southern Denmark, Odense, Denmark

## Abstract

**Background:**

The guinea pig is an attractive model for human pregnancy and placentation, mainly because of its haemomonochorial placental type, but is rather small in size. Therefore, to better understand the impact of body mass, we studied placental development in the capybara which has a body mass around 50 kg and a gestation period of around 150 days. We paid attention to the development of the lobulated arrangement of the placenta, the growth of the labyrinth in the course of gestation, the differentiation of the subplacenta, and the pattern of invasion by extraplacental trophoblast.

**Methods:**

Material was collected from six animals at pregnancy stages ranging from the late limb bud stage to mid gestation. Methods included latex casts, standard histology, immunohistochemistry for cytokeratin, vimentin, alpha-smooth muscle actin, and proliferating cell nuclear antigen as well as transmission electron microscopy.

**Results:**

At the limb bud stage, the placenta was a pad of trophoblast covered by a layer of mesoderm from which fetal vessels were beginning to penetrate at folds in the surface. By 70 days, the placenta comprised areas of labyrinth (lobes) separated by interlobular areas. Placental growth resulted predominantly from proliferation of cellular trophoblast situated in nests at the fetal side of the placenta and along internally directed projections on fetal mesenchyme. Additional proliferation was demonstrated for cellular trophoblast within the labyrinth.

Already at the limb bud stage, there was a prominent subplacenta comprising cellular and syncytial trophoblast with mesenchyme and associated blood vessels. At 90 days, differentiation was complete and similar to that seen in other hystricognath rodents. Overlap of fetal vessels and maternal blood lacunae was confirmed by latex injection of the vessels. At all stages extraplacental trophoblast was associated with the maternal arterial supply and consisted of cellular trophoblast and syncytial streamers derived from the subplacenta.

**Conclusion:**

All important characteristics of placental development and organization in the capybara resembled those found in smaller hystricognath rodents including the guinea pig. These features apparently do not dependent on body size. Clearly, placentation in hystricognaths adheres to an extraordinarily stable pattern suggesting they can be used interchangeably as models of human placenta.

## Background

Rodents are useful models for human reproduction due to the ready availability of laboratory animals [1] and their closeness to the primate lineage [2-4]. Although four suborders are recognized, most species used in research are myomorph rodents [1]. A notable exception is the guinea-pig, which is a hystricognath rodent from the suborder Hystricomorpha [5]. The hystricognath rodents have adopted a reproductive strategy characterized by a relatively long gestation, small litter size and the delivery of well-developed (precocial) young [6]. This is in many respects similar to reproduction in higher primates [7]. For this reason among others [1,8], they offer more satisfactory models for human pregnancy than rodents that have short pregnancies and deliver large litters of poorly developed (altricial) young. As an example, events occurring during later stages of pregnancy in humans must be studied postnatally in rats and mice, introducing a wealth of confounding factors. There are several similarities in placentation between hystricognaths and higher primates including a single layer of syncytiotrophoblast in contact with the maternal blood space (i.e. haemomonochorial) as opposed to three trophoblast layers (i.e. haemotrichorial) in myomorph rodents. There are as well similar patterns of trophoblast invasion and placental growth [1,9-13].

Current concepts of palaeogeography favour an African origin for hystricognaths with dispersal to South America by a trans-Atlantic route in the Eocene or Oligocene [14]. The subsequent radiation resulted in the wide range of forms found in South America today [15,16]. The semi-aquatic capybara (*Hydrochaeris hydrochaeris*) is by far the largest extant species of rodent. Like other hystricognaths, it delivers precocial neonates after a relatively long gestation period [17,18].

Although the guinea pig is an attractive model for human pregnancy, the question arises whether it is possible to compare such a small animal with the condition in humans. To better understand this we have studied placental development in the capybara, which more closely approximates human dimensions with a maternal body mass around 50 kg, a delivery weight of around 1 kg and a gestation period of around 150 days [18]. The main aim of the study is to substantiate if the principle processes of placentation depend on body size or not. Special attention was paid to the following questions: How is the lobulated arrangement of the placenta developed in the capybara? Previous studies had shown only the architecture of the term placenta [19-21]. Does the labyrinth continue to grow in the course of gestation in the same way as in smaller hystricognaths? How do the ontogenetic differentiation of the subplacenta and the associated pattern of trophoblast invasion occur? These are both specialized features of hystricognath placentation. Finally, what is the significance of these findings on placental differentiation in the capybara for the choice of smaller species as models for human placentation?

## Methods

### Tissue collection and fixation

The observations are based on material collected from six animals at various stages of pregnancy (Table 1). Relevant placental characteristics of the capybara and related hystricognath species investigated so far are summed up in Tables 2 and 3[6,9-13,19-50].

**Table 1:** Fetal and placental size at the four stages of gestation studied

Crown-rump length of fetus (cm)	Placental size (cm)^a^	Estimated gestational age (days) [18]	Number of placentas studied
1.2 (n = 1)	1.5 × 1.3	Late limb bud stage	1
5.5–5.8 (n = 2)	3.5 × 2.5	70	2
8.0–13.0 (n = 3)	6.7 × 4.0	90	4

^a^Greatest diameter × greatest depth (including subplacenta).

**Table 2:** List of major placental characters and conditions in Hystricognathi

Nr.	Character	Character condition(s)
1	Organization of main placenta	Moderate lobulation (1). Complex lobulation (2).
2	Capsule around placenta	Absent (1). Present until mid gestation (2).
3	Labyrinth I	Radial arrangement of the exchange areas around the maternal blood lacunas (1).
4	Labyrinth II	Countercurrent arrangement of the fetal and maternal blood flows (1).
5	Interhaemal barrier	Haemochorial placenta with cellular and syncytial trophoblast in early ontogeny and mostly syncytial barrier later on (1).
6	Interlobium	Substantial areas of interlobium associated with labyrinth (1).
7	Placental growth	Essentially the result of specialised growing zones at the outer margin, associated with internally directed projections on fetal mesenchyme (1).
8	Additional proliferation	Insignificant proliferation activity in trophoblast in the labyrinth (1). Remarkable proliferation in this trophoblast in early and mid gestation (2).
9	Presence of subplacenta	Distinct and specialised area, consisting of layers of cellular and syncytial trophoblast on fetal mesenchyme, occurring from early ontogeny to near term or term (1).
10	Blood supply of subplacenta	Associated with the maternal circulation in early ontogeny and supplemented by the fetal system later, without overlap between the two systems (1). Same composition, but some overlap between the systems during mid gestation (2).
11	Extraplacental trophoblast	Both cellular trophoblast and syncytial streamers in the decidua between the subplacenta and the materal arterial system, probably responsible for the replacement of the maternal arterial endothelium (1).
12	Visceral yolk sac	Inverted yolk sac with conspicuous villous areas (1).
13	Fibrovascular ring	Specialised arterial and capillary system within the yolk sac near the attachment to the main placenta (1).
14	Parietal yolk sac	Present, usually multi-layered from mid gestation on (1).

**Table 3:** Distribution of placental characteristics in hystricognath rodents

Taxon	1	2	3	4	5	6	7	8	9	10	11	12	13	14	Principle source
Capybara *(Hydrochaeris)*	2	2	1	1	1	1	1	2	1	2	1	1	1	1	[19-21], own data
Guinea pig *(Cavia)*	2	1	1	1	1	1	1	2	1	1	1	1	1	1	[6,9-12,21,27,32,33,38-41]
Prea (*Galea*)	2	1	1	1	1	1	1	2	1	2	1	1	1	1	[30,31]
Rock cavy *(Kerodon)*	2	1	1	1	1	1	1	2	1	1	1	1	1	1	[28], own data
Paca *(Cuniculus)*	2	2	1	1	1	1	1	2	1	1	1	1	1	1	[20,21,42], own data
Agouti *(Dasyprocta)*	2	2	1	1	1	1	1	2	1	1	1	1	1	1	[20,21,30,34,42], own data
Chinchilla *(Chinchilla)*	2	1	1	1	1	1	1	?	1	1	1	1	1	1	[26,43,44]
Degu *(Octodon)*	1	1	1	1	1	1	1	1	1	2	1	1	1	1	[6,9,12,13,35-37,45,46]
Nutria *(Myocastor)*	2	2	1	1	?	1	1	?	1	1	1	1	1	1	[25,47]
Canadian porcupine (*Erethizon)*	2	1	1	1	1	1	?	?	1	1	1	1	1	1	[24]
Dassie rat *(Petromus)*	1	1	1	1	1	1	1	1	1	1	1	1	1	1	[6,9,29,35,45]
Cane rat *(Thryonomys)*	2	1	1	1	1	1	1	?	1	1	?	1	1	1	[6,48-50]
African porcupine *(Hystrix)*	2	1	1	1	1	1	1	?	1	1	1	1	1	1	[22]
Mole rat *(Bathyergus)*	2	1	1	1	1	1	1	?	1	1	1	1	1	1	[22]

The principle sources and the various character conditions are given. See Table 2 for a description of characters and character conditions. Unknown conditions are referred to by a question mark. The capybara, guinea pig, prea and rock cavy are members of the same family (Caviidae). Each of the other species represents a different family.

Capybara material was collected at hysterectomy from animals bred at the Centre for Experimental Breeding of Capybaras, Paulista State University, Araçatuba, São Paulo, as authorized by the Brazilian Institute of the Environment and Renewable Natural Resources (IBAMA). Additional material (n = 3) was obtained at an IBAMA licensed slaughterhouse (Panamby-Porã, Miracatu, S.P.). The experimental protocol was approved by the Bioethics Committee of the School of Veterinary Medicine, University of Sao Paulo.

Bilateral hysterectomy was performed in 3 capybaras. The animals were premedicated with acepromazine (Univet, São Paulo, S.P., Brazil; 0.1–1.0 mg/kg I.M.). Anaesthesia was induced with xylazine (Dorcipec^®^, Vallée S.A., Montes Claros, M.G., Brazil; 0.5–1.0 mg/kg) and ketamine (Cristália, Itapira, S.P., Brazil; 5–10 mg/kg I.M.) and continued with halothane (Hoechst, Frankfurt, Germany; 1 per cent) or enflurane (Etrane^®^, Abbott, São Paulo, S.P., Brazil) in oxygen. Postoperative treatment included antibiotic coverage with benzyl penicillin and streptomycin (Pentabiotico^®^, Fort Dodge, Campinas, S.P., Brazil; 8000–24 000 IU/kg I.M.) and analgesia as required with flunixin meglumine (Banamine^®^, Schering-Plough, Rio de Janeiro, R.J., Brazil).

In one placenta from mid gestation the maternal and fetal vessels were injected with coloured latex (uterine artery white, uterine vein blue, umbilical artery yellow and umbilical vein red) in order to show the vessel distribution.

Tissues collected for histology and immunohistochemistry were immersion fixed in 10 per cent formalin in 0.1 M phosphate buffer, pH 7.4, for 24–48 h. After fixation they were submitted to dehydration and embedded in paraplast. Tissues for transmission electron microscopy were fixed in 2.5% glutaraldehyde or 2% paraformaldehyde/2.5% glutaraldehyde for 24 h and embedded in araldite as described below or in Spurr?s resin.

### Histology and immunohistochemistry

The blocks were sectioned at 5 μm using an automatic microtome (Leica RM2155, Germany). Sections were stained by standard procedures with haematoxylin and eosin, Masson's trichrome and the periodic acid-Schiff reaction (PAS).

Following an approach established by Carter at al. [51], immunohistochemistry was performed for cytokeratin to identify epithelial cells and trophoblasts; vimentin to identify mesenchymal cells and stromal decidua; and α-smooth muscle actin to identify vessel walls. As a proliferation marker we used a mouse monoclonal antibody to human proliferating cell nuclear antigen (PCNA).

Sections were dewaxed then rehydrated in an ethanol series and in the course of this they were submitted to endogenous peroxidase blockage in 3% hydrogen peroxide (v/v) in ethanol for 20 minutes. They were then placed in 0.1 M citrate buffer, pH 6.0, and submitted to microwave irradiation at 700 MHz for fifteen minutes. The sections were equilibrated in 0.1 M phosphate-buffered saline (PBS), pH 7.4, and non-specific binding was blocked using Dako Protein Block (DakoCytomation, Carpinteria, California, USA) for 20 minutes.

Tissues were incubated with primary antibodies overnight at 4°C in a humid chamber. Cytokeratin was detected by a rabbit polyclonal antibody (1:500; PU071-UP, Biogenex, San Ramon, California, U.S.A.). Mouse monoclonal anti-human primary antibodies were used to detect vimentin (1:200; V9, sc-6260, Santa Cruz Biotechnology, Santa Cruz, California, USA), α-smooth muscle actin (1:300; Clone 1A4, DakoCytomation, Carpinteria, California, USA), and PCNA (1:100; PC10, sc-56, Santa Cruz Biotechnology, Santa Cruz, California, USA). The slices were then rinsed in PBS and incubated with the biotinylated secondary antibody for 45 minutes, followed by streptavidin-HRP for 45 minutes (LSAB^®^+ System-HRP, DakoCytomation, Carpinteria, California, USA). After rinsing in PBS, the binding was visualized using aminoethyl carbazole (AEC Substrate Kit, Zymed Laboratories, South San Francisico, California, USA) or diaminobenzidine (DAB) as the chromagen. The sections were counterstained with haematoxylin and mounted in Faramount^® ^(DakoCytomation, Carpinteria, California, USA) or Permount^® ^(Fisher Scientific, Fair Lawn, New Jersey, USA). Negative controls were performed using PBS instead of primary antibody solution.

### Transmission electron microscopy

Post fixation was in 2% phosphate-buffered osmium tetroxide, pH 7.4, for 2 h. Tissues were then washed in PBS (3 × 10 min) and immersed in a saturated uranyl acetate solution for 1 h. After washing in distilled water (3 × 10 min), they were dehydrated in alcohol and immersed in propylene oxide for 15 min. They were then immersed in a 2:1 mixture of propylene oxide and araldite (Polysciences Inc., Warrington, Pennsylvania, USA) for 1 h, in a 1:1 mixture for 30 min, in a 1:2 mixture for 2.5 h and in araldite for 3 h. Finally, they were embedded in araldite and baked in an oven at 70°C for 2–3 days to complete polymerization.

Semithin sections were cut at 1 μm on an automatic ultramicrotome (Ultracut R, Leica Microsystems, Nussloch, Germany) and stained with a 1% aqueous solution of toluidine blue to identify areas of interest. Ultrathin sections 70 nm thick were collected on copper mesh and contrasted with 2% uranyl acetate for 7–10 min and with 0.5% lead citrate for 7–10 min. Finally, the sections were studied in a transmission electron microscope (Morgagni 268D, FEI Company, Eindhoven, the Netherlands). Images were captured with a MegaView III camera linked to an image analysis system (Soft Imaging System, Münster, Germany).

## Results

### General description

**Figure 1:**
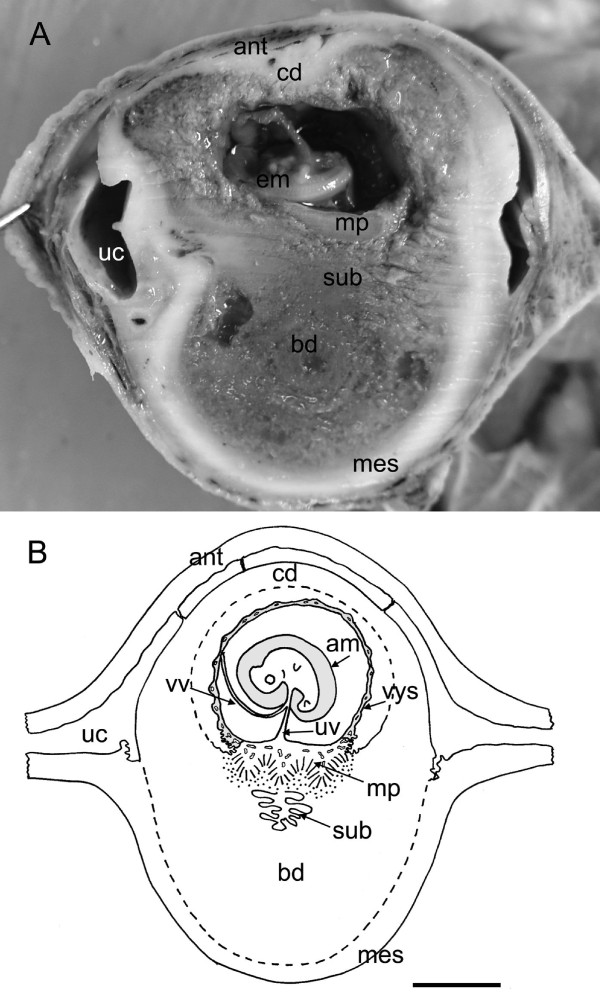
**Implantation site in the capybara at the limb bud stage of development**. (A) Median sagittal section of capybara implantation site at the limb bud stage of development. (B) Schematic drawing of the same stage. The embryo and membranes are enclosed within the uterine wall. A thick capsular decidua faces the antimesometrial wall of the uterus. The main placenta and subplacenta are attached to the basal decidua on the mesometrial side. Membranes include the amnion and visceral yolk sac. The latter is supplied by vitelline vessels whilst umbilical vessels supply the placenta. The uterine cavity is also shown. Am = amnion, ant = antimesometrial wall of the uterus, bd = basal decidua, cd = capsular decidua, em = embryo, mes = mesometrial side, mp = main placenta, sub = subplacenta, uc = uterine cavity, vys = visceral yolk sac, vv = vitelline vessels. Scale bar = 1 cm.

**Figure 2:**
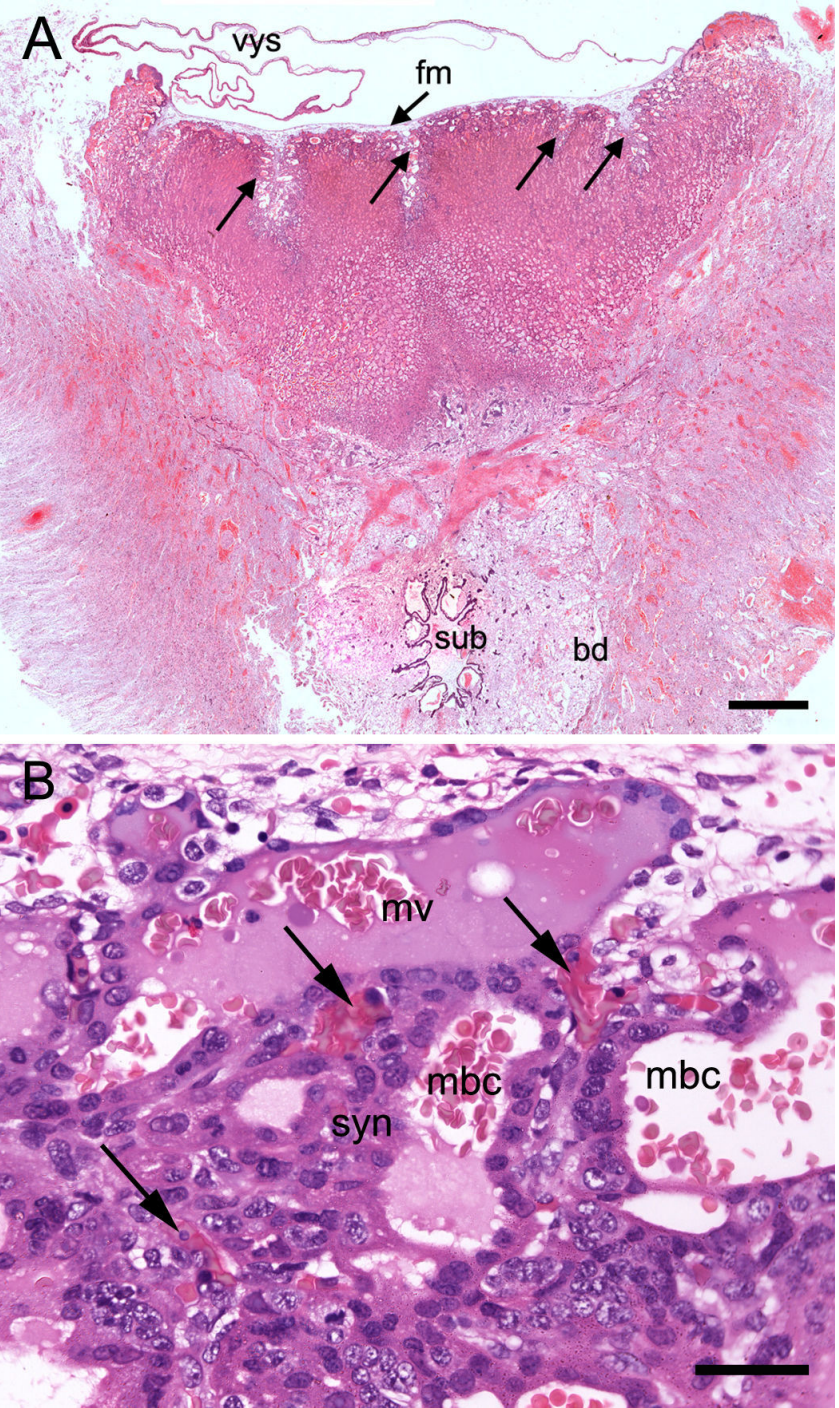
**Placentation in the capybara at the limb bud stage**. (A) The chorioallantoic placenta is a pad of trophoblast covered by allantoic (fetal) mesoderm from which vessels are beginning to penetrate the trophoblast at several folds (arrows). The subplacenta is situated deep within the basal decidua. The visceral yolk sac is inserted on the marginal surface of the placenta and is largely unfolded. Haematoxylin and eosin. (B) The interhaemal membrane is haemomonochorial from an early stage. A superficial maternal vessel is seen from which blood enters maternal blood spaces separated from fetal capillaries (arrows) by syncytiotrophoblast. Haematoxylin and eosin. Bd = basal decidua, fm = allantoic or fetal mesoderm, mbc = maternal blood space, mv = maternal vessel, sub = subplacenta, syn = syncytiotrophoblast, vys = visceral yolk sac. Scale bars = 1.5 mm (A), 40 μm (B).

Six placentas were processed for histology and three for transmission electron microscopy (Table 1). At the earliest stage available, a late limb bud stage, the embryos were deep in the decidua and covered by a thick capsule (Figure 1A–B, Table 2 and 3, character 2). The five embryos had an average crown-rump length of 11.5 mm. The forelimb and hind limb buds were present, with the forelimbs being larger and close to the somites. The cervical flexure was evident. The chorioallantoic placenta was a pad of trophoblast (Figure 2A) covered by a layer of allantoic mesoderm from which fetal vessels were just beginning to penetrate the trophoblast at folds in the surface. There was a prominent subplacenta that occupied about half the total depth of the placenta (Figure 2A). It was situated deep in the decidua and associated with the maternal vasculature (Table 2 and 3, characters 9, 10). The yolk sac was inserted on the marginal surface of the placenta (Table 2 and 3, character 12) and is described below.

**Figure 3:**
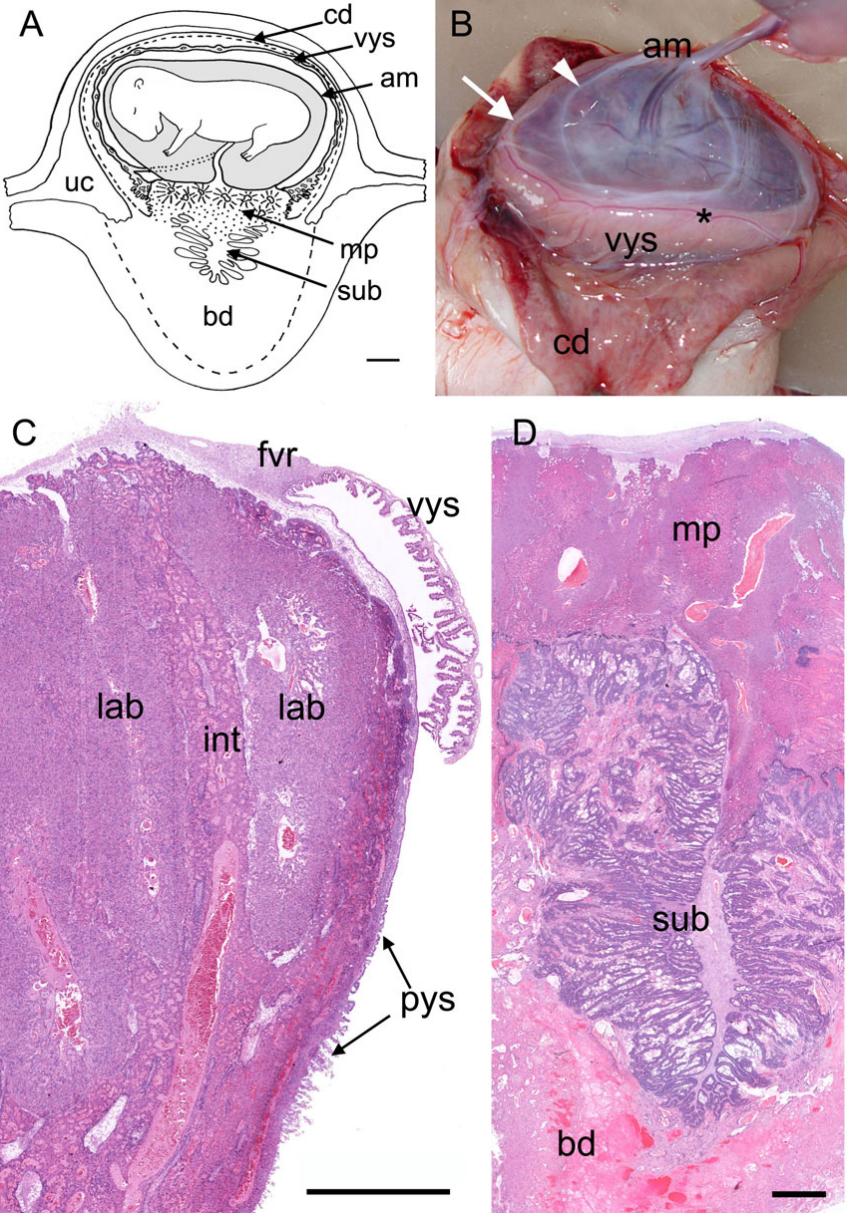
**Placentation in the capybara at around 70 days of gestation**. (A) Schematic drawing showing the thin capsular decidua, visceral yolk sac, amnion, main placenta, subplacenta and basal decidua. (B) The capsular decidua has been opened and reflected to expose the fetal membranes. The amnion has also been cut open but retains its attachment to the surface of the chorioallantoic placenta (arrowhead). The visceral yolk sac is inserted more peripherally (arrow). The yolk sac vessels terminate in the sinus terminalis (asterisk). (C) At this stage the lobes of labyrinth are clearly separated by interlobar areas. This view also shows the relation between the visceral and parietal parts of the yolk sac, the fibrovascular ring and the margin of the chorioallantoic placenta. Haematoxylin and eosin. (D) The subplacenta is fully developed and is situated below the main placenta and within the basal decidua. Haematoxylin and eosin. Am = amnion, bd = basal decidua, cd = capsular decidua, fvr = fibrovascular ring, int = interlobar areas, lab = labyrinth, mp = main placenta, pys = parietal yolk sac, sub = subplacenta, uc = uterine cavity, vys = visceral yolk sac. Scale bars = 1 cm (A), 1.5 mm (C-D).

At the next stage, estimated at 70 days gestation [18], the capsule was present as a thin membrane (Figure 3A–B). Differentiation of the placenta into areas of labyrinth was well advanced (Figure 3C) and these areas were separated by bands of trophoblast without fetal capillaries, the interlobular trophoblast (Table 2 and 3, character 1). The areas of labyrinth extended from near the surface of the placenta. There was a substantial amount of trophoblast beneath them that still was not differentiated into labyrinth or interlobium. The subplacenta was fully developed (Figure 3D) and extraplacental trophoblast could be followed from this organ into the decidua (Table 2 and 3, character 11). The yolk sac was largely villous except for the fibrovascular ring at the attachment to the placental disk (Figure 3C, Table 2 and 3, characters 12, 13).

**Figure 4:**
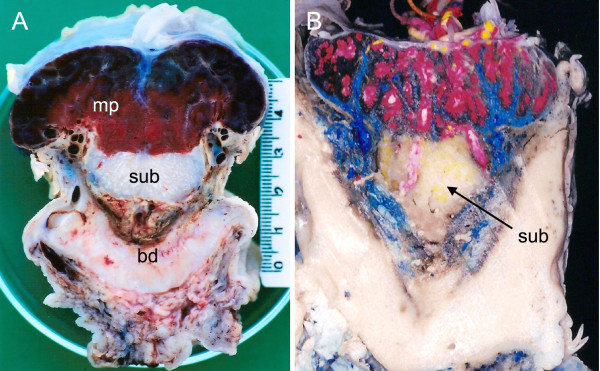
**Placentation in the capybara at around 90 days of gestation**. (A) Median sagittal section through a fresh placenta to show the extent and appearance of the main placenta, subplacenta and basal decidua. (B) Median sagittal section through a placenta following injection of coloured latex in the uterine artery (white), uterine vein (blue), umbilical artery (yellow) and umbilical vein (red). Maternal and fetal vessels are seen in the subplacenta. Bd = basal decidua, mp = main placenta, sub = subplacenta.

By the following stage, estimated at 90 days gestation [18], the capsule no longer covered the conceptus and the visceral yolk sac was exposed to the uterine lumen. The placenta now had the lobulated appearance typical of the hystricognath placenta, with some of the lobes far from the surface and surrounded by interlobular trophoblast (Figure 4A–B, Table 2 and 3, characters 1 to 6). The subplacenta remained about the same size whereas the disk above it had continued to grow.

### Growth of the main placenta

#### Limb bud stage

**Figure 5:**
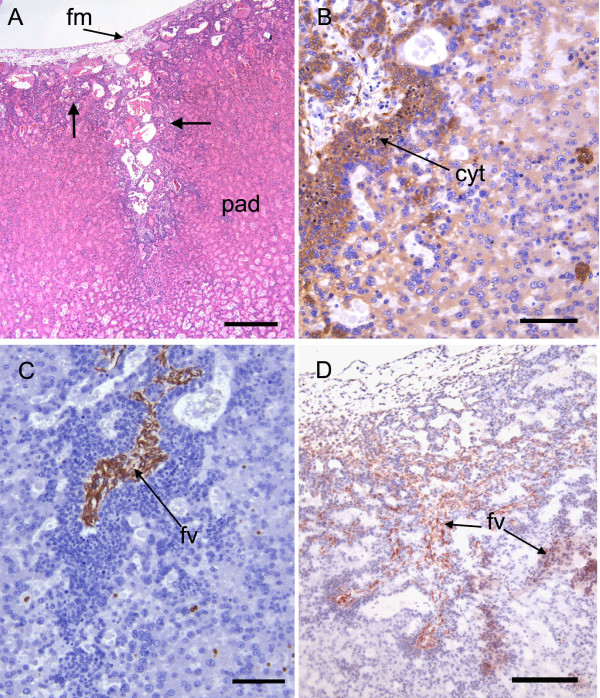
**Chorioallantoic placenta of the capybara at the limb bud stage I**. (A) An extensive pad of spongy trophoblast is covered on the fetal surface by allantoic mesoderm. Note the cytotrophoblasts that occur at the surface and along the margins of the fold (arrows). Haematoxylin and eosin. (B) Immunostaining for cytokeratin is more intense in the cytotrophoblasts lining the fold than in the rest of the trophoblast pad. (C) Immunostaining for vimentin confirms the presence of fetal vessels within a fold. (D) Immunostaining for smooth muscle α-actin reveals that fetal vessels are beginning to ramify into the trophoblast at the margin of a fold. Cyt = cytotrophoblasts, fm = allantoic or fetal mesoderm, fv = fetal vessel, pad = trophoblast pad. Scale bars = 500 μm (A), 100 μm (B-C), 200 μm (D).

**Figure 6:**
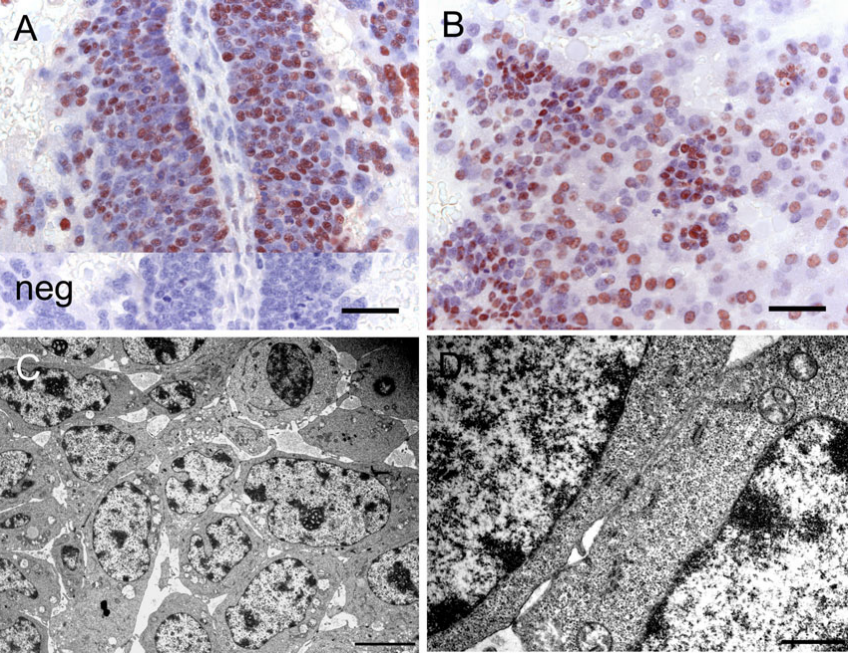
**Chorioallantoic placenta of the capybara at the limb bud stage II**. (A) Immunostaining for PCNA indicates proliferation of the cytotrophoblasts lining a fold. Inset is a negative control (neg). (B) Immunostaining for PCNA also reveals nests of cytotrophoblasts within the spongy trophoblast pad. (C) Ultrastructure of cytotrophoblasts. TEM. (D) Detail of the cytotrophoblasts. TEM. Scale bars = 40 μm (A-B), 5 μm (C), 1 μm (D).

At this stage, most of the trophoblast formed a spongy pad containing maternal blood spaces (Figure 2A). The centre of the disk was covered on the fetal side by connective tissue (allantoic mesoderm) containing the fetal blood vessels. On the fetal side of the pad there were large nests of cellular trophoblast (Figure 5A–B). More centrally the pad consisted of both cellular and syncytial trophoblast. In addition spurs of connective tissue lined by cytotrophoblasts passed into the pad both in the region of the central excavation and more laterally. The incipient vascularization of the placenta by vessels emanating from these folds of connective tissue could be confirmed by immunostaining for vimentin and actin (Figure 5C–D). The nuclei of the cytotrophoblasts were shown to have high proliferation activity by immunostaining for PCNA (Figure 6A). In addition positive records for immunostaining for PCNA occurred within the spongy trophoblast pad (Figure 6B). At the ultrastructural level (Figure 6C–D), we saw nests of cellular trophoblast with several mitotic figures and large intercellular spaces (Table 2 and 3, character 7).

#### Gestational age 70 days

**Figure 7:**
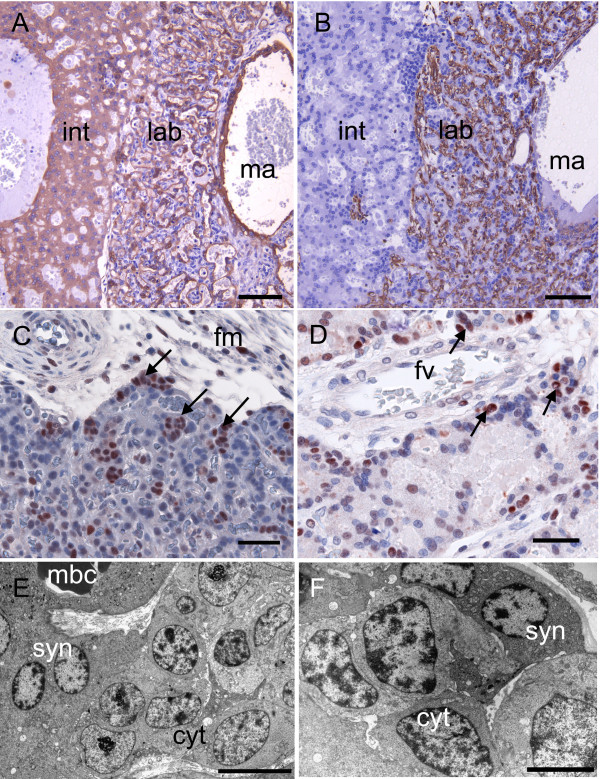
**Chorioallantoic placenta of the capybara at around 70 days of gestation**. (A) Labyrinth and interlobular area of capybara placenta immunostained for cytokeratin. Note the strong reaction of trophoblast lining a large maternal arterial channel at the center of the lobe. (B) Similar area immunostained for vimentin, which picks out the capillaries of the labyrinth. (C) Immunostaining for PCNA reveals nests of proliferative cytotrophoblast (arrows) underlying allantoic mesoderm at the centre of the placental disk. (D) Immunostaining for PCNA shows that similar, proliferative cytotrophoblast occurs in the labyrinth especially close to the strand of mesoderm carrying the fetal vessel. (E) A nest of proliferative cytotrophoblast is accompanied by a layer of syncytiotrophoblast that lines a maternal blood channel.TEM. (F) Detail of cellular and syncytial trophoblast in this region. TEM. Cyt = cytotrophoblasts, fm = allantoic or fetal mesoderm, fv = fetal vessel, int = interlobar areas, lab = labyrinth, ma = maternal arterial channel, mbc = maternal blood channel, syn = syncytiotrophoblast. Scale bars = 100 μm (A-B), 40 μm (C-D), 10 μm (E) 5 μm (F).

By this stage the main placenta was composed of areas of labyrinth, or lobes, with interlobular areas between them (Figures 3C, 7A–B). Large arterial blood channels lined with trophoblast were found at the centre of each lobe (Figure 7A). The labyrinth consisted of maternal blood spaces lined by syncytial trophoblast and fetal capillaries that were immunopositive for vimentin (Figure 7B). The maternal blood spaces could be seen to radiate from the larger blood channels at the centre of the lobe (Table 2 and 3, characters 3, 4). The interlobular areas between the placental lobes had a different organization. These areas were mostly composed of syncytiotrophoblast. The syncytial nuclei were dispersed with euchromatin and were spherical in shape. The cytoplasm was more eosinophilic than in the labyrinth. Within the syncytial mesh were maternal venous blood channels. However, nests of proliferating cytotrophoblast were still present at the fetal border of the placenta (Figure 7C) and along the strands of fetal mesoderm that carried fetal vessels towards the labyrinth (Figure 7D). Proliferating cellular trophoblast was even present within the labyrinth (not shown, Table 2 and 3, character 8). As at the stage described above, large intercellular spaces occurred between the cytotrophoblasts as well as between them and the layer of syncytiotrophoblast that lined the maternal blood channels (Figure 7E–F, Table 2 and 3, character 5).

#### Gestational age 90 days (mid term)

**Figure 8:**
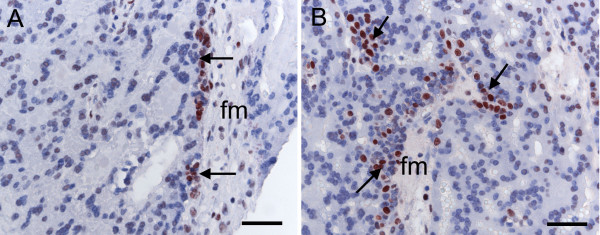
**Chorioallantoic placenta of the capybara at around 90 days of gestation**. (A) Immunostaining for PCNA shows proliferative cytotrophoblast (arrows) under the allantoic mesoderm near the surface of the placenta. (B) Proliferating cells (arrows) are still seen within the placenta both in association with fetal mesoderm and within the labyrinth. Fm = allantoic or fetal mesoderm. Scale bars = 40 μm.

The labyrinth was fully organized by mid gestation with fetal capillaries running parallel to maternal blood channels in a countercurrent arrangement (data not shown). The proliferation marker still revealed nests of proliferating cytotrophoblast near the surface of the placenta, associated with fetal mesoderm in deeper layers and within the labyrinth itself (Figure 8A–B).

### Subplacenta

#### Limb bud stage

**Figure 9:**
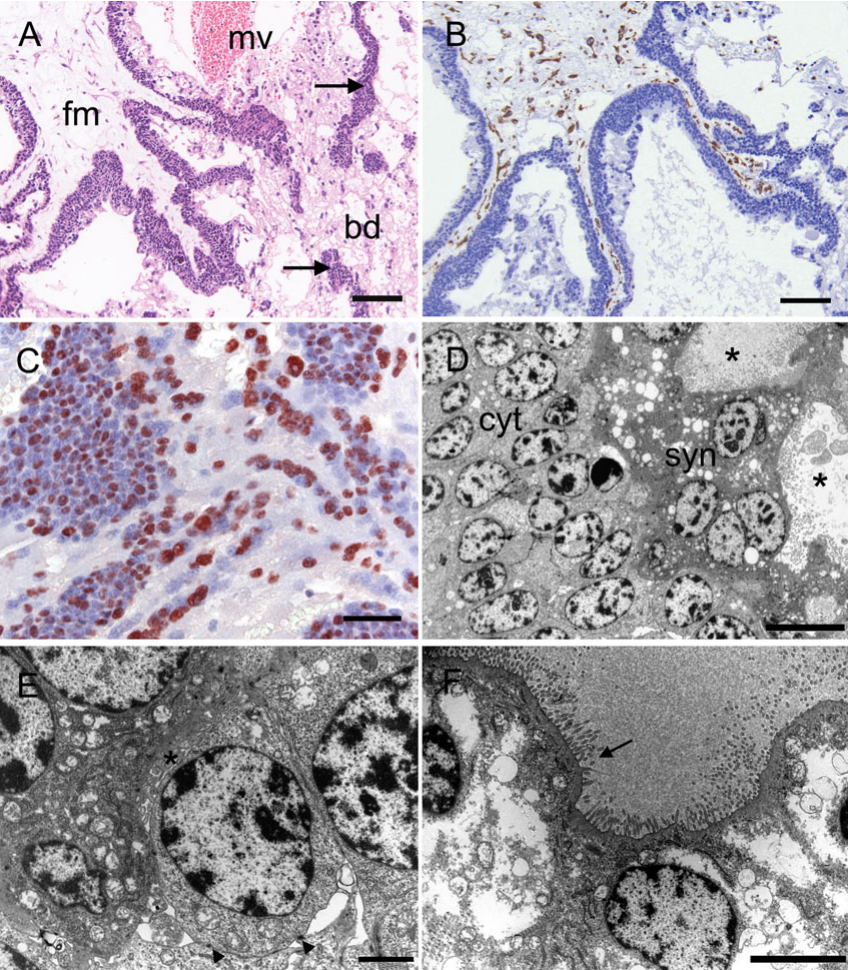
**Subplacenta of the capybara at the limb bud stage**. (A) Cytotrophoblast lines the fetal mesenchyme of the central excavation but also extends (arrows) into the surrounding basal decidua. Note the close proximity of a large maternal vessel. Haematoxylin and eosin. (B) In a similar field immunostaining for vimentin picks out the fetal blood vessels of the subplacenta. (C) Immunostaining for PCNA shows that the cytotrophoblast of the subplacenta is highly proliferative. (D) The cytotrophoblast is multilayered. The syncytiotrophoblast contains vacuoles and sends microvilli into extracellular spaces (asterisks). TEM. (E) Desmosomes (arrowheads) are seen between adjacent cytotrophoblast cells. The surface of the syncytiotrophoblast is folded where it abuts the extracellular space (asterisk). TEM. (F) The syncytiotrophoblast, with its covering of microvilli (arrows), borders the maternal blood spaces. Large extracellular spaces are present. TEM. Bd = basal decidua, cyt = cytotrophoblasts, fm = allantoic or fetal mesoderm, mv = maternal vessel, syn = syncytiotrophoblast. Scale bars = 100 μm (A-B), 40 μm (C), 10 μm (D), 2 μm (E), 5 μm (F).

At this early stage the subplacenta was situated deep in the decidua (Figure 2A) and associated with maternal vasculature (Figure 9A). It consisted of several layers of cellular and syncytial trophoblast with fetal mesenchyme and associated blood vessels separating the various lobes (Figure 9B). The cytotrophoblasts were highly proliferative (Figure 9C). At the ultrastructural level (Figure 9D–F), desmosomes were seen between adjacent trophoblast cells. The syncytiotrophoblast contained many vacuoles and its surface was covered with microvilli that projected into large extracellular spaces. Some of these spaces contained maternal blood cells.

#### Gestational age 70 days and 90 days (mid term)

**Figure 10:**
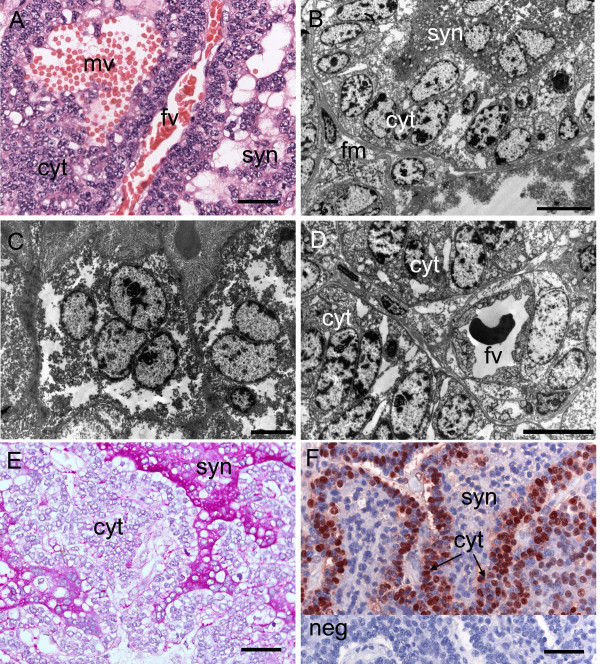
**Subplacenta at around 70 (A-E) and 90 (F) days of gestation**. (A) Note the syncytiotrophoblast and the presence in the subplacenta of a maternal vessel, which is separated by several layers of cytotrophoblast from a fetal vessel. Haematoxylin and eosin. (B) Cytotrophoblast, mostly single layered, is associated with fetal mesenchyme. On the other side of this layer, syncytiotrophoblast is found. TEM. (C) The syncytiotrophoblast contains spaces that convey a fragmented appearance. TEM. (D) A fetal vessel within the mesenchyme of the subplacenta. TEM. (E) PAS reaction is strong within the syncytiotrophoblast. (F) Immunostaining for PCNA shows that the cytotrophoblast of the subplacenta remains highly proliferative at mid gestation. Inset: negative control (neg). Cyt = cytotrophoblasts, fm = allantoic or fetal mesoderm, fv = fetal vessel, mv = maternal vessel, syn = syncytiotrophoblast. Scale bars = 40 μm (A, E, F), 10 μm (B, D), 5 μm (C).

From 70 days onwards the subplacenta was more fully differentiated (Figure 3D). Fetal vessels were found within the fetal mesenchyme, adjacent to the layer of cellular trophoblast. In addition, maternal blood lacunae were still present facing the syncytial trophoblast (Figure 10A, Table 2 and 3, character 10). Much of the cytotrophoblast was in a single layer (Figure 10B). It was lined on one side by a band of fetal mesenchyme and on the other by a layer of syncytiotrophoblast. The cytoplasm of the syncytiotrophoblast contained spaces that gave it a fragmented appearance (Figure 10C). Fetal vessels within the mesenchyme were in close proximity to the cytotrophoblast (Figure 10D). There was a strong PAS positive reaction in the syncytiotrophoblast consistent with the presence of glycogen and/or glycoprotein (Figure 10E). The cytotrophoblast remained highly proliferative at mid gestation (Figure 10F).

Even at mid gestation both fetal arteries and maternal blood lacunae were present in the subplacenta as could be confirmed by injection of the vessels with latex (Figure 4B, Table 2 and 3, character 10).

### Junctional region and decidua

#### Limb bud stage

**Figure 11:**
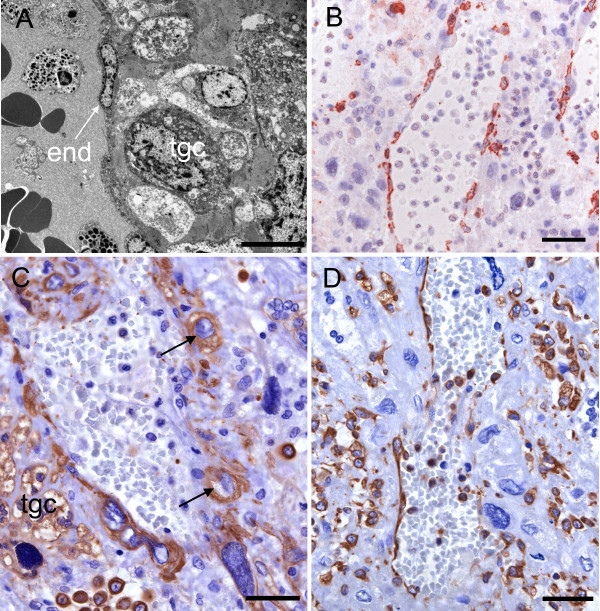
**Junctional region and decidua at limb bud stage (A-B) and 70 days of gestation (C-D)**. (A) At the limb bud stage maternal vessels in the decidua retain most of their endothelium. There is, however, intrusion of a trophoblast giant cell into the decidua subjacent to the vessel wall. TEM (B) Immunostaining for actin confirms that vessel architecture is largely intact. (C) Immunostaining for cytokeratin at 70 days reveals the presence of trophoblast cells in the vessel walls and surrounding decidua. These include giant cells, large cells (arrows) and smaller cells. (D) Immunostaining for vimentin shows a discontinuous vessel endothelium. Decidual cells also are vimentin positive. End = endothelium, tgc = trophoblast giant cell. Scale bars 10 μm (A), 40 μm (B-D).

At the ventral and lateral borders of the subplacenta, extraplacental trophoblast was evident, consisting of large cellular trophoblast and syncytial streamers. Near the subplacenta, extraplacental trophoblast had started to rebuild the walls of maternal arteries, although remnants of the vessel endothelium were still present (Figure 11A–B, Table 2 and 3, character 11).

#### Gestational age 70 days and 90 days (mid term)

At 70 days the subplacenta was associated with prominent areas of extraplacental trophoblast that could be followed deeply within the decidua. This included large and small trophoblast cells (extraplacental cytotrophoblast) and groups of trophoblast giant cells (Figure 11C) as well as strands of syncytiotrophoblast or syncytial streamers. These trophoblast cells were responsible for the destruction and replacement of the maternal vessel walls (Figure 11D). At 90 days this region was very similar except for thinning of the decidua consequent on fetal growth.

### Parietal and visceral yolk sac

#### Limb bud stage

**Figure 12:**
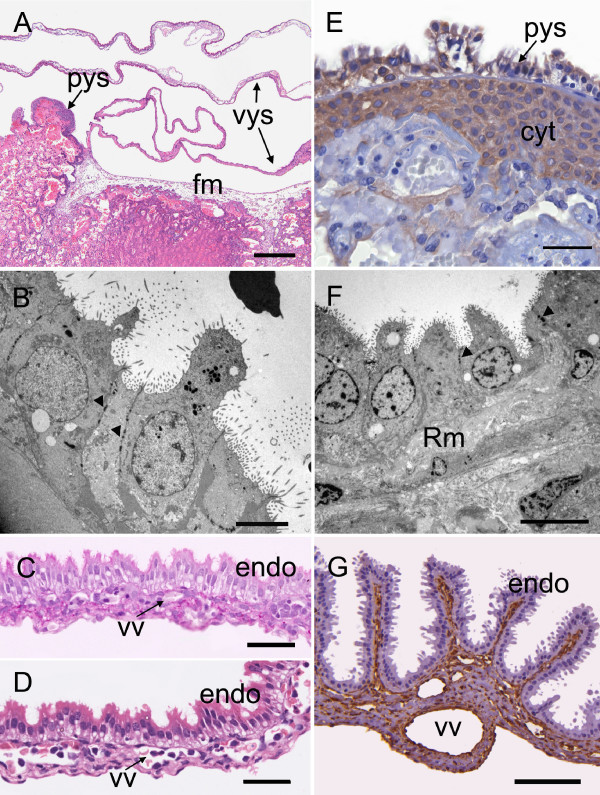
**Parietal and visceral yolk sac at limb bud stage (A-D) and 70 days of gestation (E-G)**. (A) Overview to show attachment of the visceral yolk sac to the placental disk. Lateral to this the parietal yolk sac extends over the surface as a thin epithelial layer. The central part of the disk is covered with allantoic mesoderm. (B) Endoderm of the parietal yolk sac at the limb bud stage Note the apical microvilli and the desmosomes (arrowheads). TEM. (C) Visceral yolk sac; PAS. Beneath the endoderm is a layer of mesoderm carrying the vitelline vessels. (D) Visceral yolk sac; haematoxylin and eosin. (E) The yolk sac epithelium rests on Reichert's membrane, which separates it from the surface layer of cytotrophoblast; immunostained for cytokeratin. (F) Endoderm of the parietal yolk sac at 70 days. The cells rest on Reichert's membrane. (G) Visceral yolk sac at 70 days; immunostained for vimentin. Note the villous appearance of the yolk sac at this stage. Cyt = cytotrophoblasts, endo = endoderm, fm = allantoic or fetal mesoderm, pys = parietal yolk sac, Rm = Reichert's membrane, vys = visceral yolk sac, vv = vitelline vessels. Scale bars 500 μm (A), 5 μm (B), 40 μm (C-E), 10 μm (F) 100 μm (G).

Rodents retain an inverted yolk sac placenta throughout gestation (Figures 3B, 12A). In the capybara at this stage the visceral yolk sac was fairly smooth (Figure 12A, E–F). It was attached to the placental disk and lateral to the attachment the endoderm continued as a thin epithelium referred to as the parietal yolk sac (Figure 12A). The cells rested on Reichert's membrane, but it was poorly developed at this time. At the ultrastructural level the lateral cell membranes were attached by desmosomes. There were numerous microvilli on the apical surface of the cells (Figure 12C).

#### Gestational age 70 days and mid term

The attachment of the visceral yolk sac to the disk now had the distinctive form of a fibrovascular ring as known from other hystricomorph rodents (Figure 3C, Table 2 and 3, character 13). In the area near the disk the visceral yolk sac was villous in structure (Figure 12G). Most of the parietal yolk sac consisted of a single cell layer separated by Reichert's membrane from the cytotrophoblast layer of the main placenta (Figure 12B). Near the base of the disk it took on a villous appearance (Figure 3C, Table 2 and 3, character 14). The ultrastructure of the cells had not changed greatly since the previous stage but Reichert's membrane was noticeably thicker (Figure 12D). By 70 days the yolk sac had achieved the form previously described in detail for the term placenta [20].

## Discussion

Among rodents, and especially within the subgroup Hystricognathi, there is an enormous variation in size. With a body weight of around 50 kg the capybara is by far the largest living rodent. Thus, the question arises of whether placental development in the capybara follows the same course as in its much smaller relatives. The capybara is the only rodent species that approximates human dimensions in body mass of mother and offspring. Our findings on placental development in the capybara are therefore a useful test of the presumed suitability of hystricognath rodents, particularly the guinea pig, as animal models for human placentation.

Firstly, how is a fetus of this size supported and how does the associated placental architecture develop? Even in rodents that deliver smaller and less well developed young, placental gas exchange is optimized by countercurrent arrangement of the maternal and fetal blood vessels. As shown by Mossman [52], no further improvement in efficiency can be gained by increasing the length of the capillaries. The solution adopted by the hystricognath rodents was to fold the labyrinth, thus increasing the exchange area while keeping the placenta quite compact. The lobulated appearance of the placenta in cross section is a result of this and has been considered a defining feature of the hystricognath placenta [9,10,21-23]. In the capybara, first steps towards this arrangement are apparent at an ontogenetic stage of around 70 days. Full establishment of the highly complex, lobulated appearance is achieved by 90 days, and is then equivalent to the condition of the term placenta [19-21]. As in the mature placenta, by mid gestation the labyrinth shows a fully organized counter current arrangement of maternal blood channels, lined by trophoblast, and fetal capillaries. This is associated with the presence of large arterial blood channels at the centre of each lobe and interlobular areas to collect the maternal blood from the lobes. Thus, establishment of the placental architecture of the capybara is similar to that described for other hystricognath species [[6,9,10,19-51]; see Tables 2 and 3]. In the capybara material investigated, ranging from a limb bud stage to a mid gestation stage of 90 days, placental growth is predominantly the result of proliferation of cellular trophoblast situated in nests at the fetal side of the placenta and along internally directed projections on fetal mesenchyme. Additional proliferation has been demonstrated for cellular trophoblast inside the labyrinth. This pattern is present in later stages, too, and continues to near term (data not shown). Thus, even though placental dimensions are much larger in the capybara, the essential growth processes are equal to those in other hystricognaths, especially those with a highly lobulated placental architecture [[11,13], Tables 2 and 3].

Secondly, a unique feature of the placenta of hystricognath rodents is the subplacenta. Its purpose is not fully understood but one function is to act as a source for the trophoblast that invades and transforms the maternal arteries [10,12]. This process is important in ensuring an adequate blood supply to the placenta and is analogous to the transformation of the spiral arteries in the human placenta [12]. As in the early development of other hystricognaths [[6,9,10,12,19-29,31-44,46-50], Tables 2 and 3], a subplacenta associated with the maternal vasculature and characterised by layers of syncytial and highly proliferative cellular trophoblast, can be confirmed at an early stage in the capybara. At mid gestation differentiation is typical of that in other hystricognaths, but there is an overlap of fetal arteries and maternal blood lacunae that could be confirmed by injection of the vessels with latex. In contrast, the subplacenta is supplied only by fetal vessels at term. The overlap between the two systems in mid gestation is a rare feature among hystricognaths (Tables 2 and 3), known only for the degu [35] and the prea [31], whereas the ancient condition of hystricognaths does not include an overlap or fetomaternal exchange inside the subplacenta [35]. All stages of placentation in the capybara that we investigated had extraplacental trophoblast, i.e. large cellular trophoblast cells and syncytial streamers derived from the subplacenta, that was responsible for the destruction and replacement of the maternal vessel walls deep within the decidua. This likewise represents a typical hystricognath feature [10,12,19,31,35-37]. Thus apart from the difference in placental diameter, both the differentiation of the subplacenta and its role for trophoblast invasion is similar within hystricognaths.

Based on this and previous studies we are able to list 14 characteristics of placentation that hold across the 11 families so far studied, including African representatives such as the cane rat, African porcupine and mole rat (Table 2). As shown in Table 3, just four of these show sufficient variation between species to justify definition of two character states. This indicates an extraordinarily stable pattern that clearly was evolved before dispersal of the group to South America [14].

## Conclusion

In summary, our findings on the capybara, a rodent with around 50 kg body mass, show that all important characteristics of placental development and organization in guinea pig related rodents are similar and do not vary with body mass. Therefore it is not necessary to solve the challenges of animal husbandry that presently preclude use of the capybara as a laboratory animal. Rather, our data indicate that its smaller relatives, especially the guinea pig, are adequate models for human placentation and pregnancy.

## Competing interests

The authors declare that they have no competing interests.

## Authors' contributions

TCS and MAM devised the study, participated in its design and coordination and helped to write the manuscript. CK and TCS performed the major part of the histological analysis. AMC and AMM participated in the study design and analysis and wrote the manuscript. All authors read and approved the final manuscript.
